# Iatrogenic hemodilution: a possible cause for avoidable blood transfusions?

**DOI:** 10.1186/s13054-017-1872-1

**Published:** 2017-11-25

**Authors:** Azriel Perel

**Affiliations:** Department of Anaesthesiology and Intensive Care, Sheba Medical Center, Tel Aviv University, Tel Aviv, 52621 Israel

**Keywords:** Anemia, Blood transfusion, Hemodilution, Hemoglobin, Fluid administration, Goal-directed therapy

A restrictive approach to blood transfusions is recommended by most current guidelines [1, 2]. Others suggest that in some patients a more liberal transfusion strategy may be beneficial [3]. However, the extensive discussions regarding the appropriate transfusion threshold have not adequately addressed the potential impact of iatrogenic hemodilution on the hemoglobin (Hb) level during dynamic clinical conditions that necessitate fluid administration [4, 5]. This editorial will attempt to describe the frequent occurrence of iatrogenic hemodilution and its potential impact on decisions to transfuse blood.

## The effect of fluid administration on the hemoglobin concentration

Fluid administration may result in a beneficial increase in microvascular flow (and perfusion pressure) with a global increase in oxygen delivery (DO2) and cellular oxygenation in conditions of relative volume deficit (e.g., distributive shock in sepsis). Fluid administration may also cause a relative, but not absolute, reduction in hemoglobin (Hb) concentration (“dilutional anemia”). Such iatrogenic hemodilution may cause a paradoxical decrease in DO2 due to the resulting decrease in Hb concentration, as observed in patients who have received more colloids as part of perioperative goal-directed therapy (GDT) [6] and in critically ill patients who did [7] or did not [8] increase their cardiac output following fluid loading. In patients in septic shock who received large amounts of fluids as part of the original early GDT protocol, a decrease of 30% in hematocrit was uniformly observed 3 h into the resuscitation, possibly explaining the very high incidence of blood transfusions in this group of patients [9]. From a very rough analysis of these and other studies in surgical and critically ill patients, it seems that the administration of 500 ml of fluids may acutely decrease the Hb concentration by about 1 g/dl, or about 8% [8]. A similar degree of hemodilution was observed in healthy volunteers in whom sequential Hb measurements were used to assess the impact of crystalloid administration [10]. It should be noted that the microcirculation of healthy individuals may respond differently to macrocirculatory derangements than that of surgical patients, and may differ further in critically ill patients with systemic inflammation.

## The potential impact of hemodilution on blood transfusions

It is well established that iatrogenic hemodilution may lead to increased blood transfusion due to dilutional coagulopathy and increased surgical bleeding. However, the resulting decrease in Hb levels may reach values below the acceptable transfusion threshold, and thus prompt clinicians to order blood transfusions in the absence of significant bleeding [4, 5]. This phenomenon should be considered as a potential unintended consequence of the administration of large amounts of fluids.

**Fig. 1:**
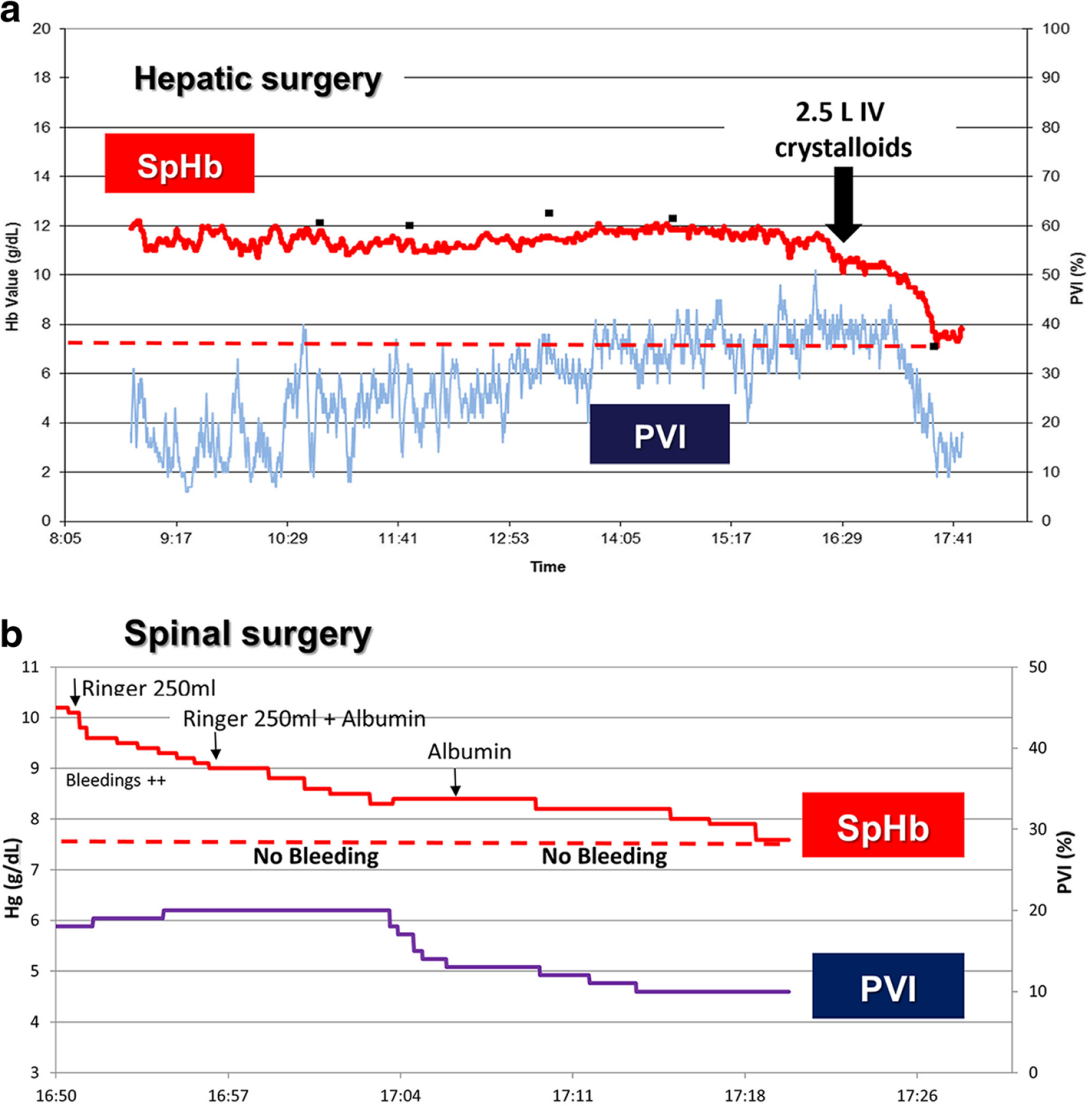
Continuous hemoglobin (SpHb) and Pleth Variability Index (PVI) during hepatic (**a**) and spinal (**b**) surgery. **a** The gradual increase in the PVI to very excessive values (close to 40%) signifies the development of hypovolemia during the hepatic resection phase. Aggressive fluid rehydration at the end of the resection phase led to the immediate decrease of the PVI and a simultaneous significant decrease in SpHb denoting acute hemodilution, which in this case probably reveals true anemia. **b** The characteristic decrease in both SpHb and PVI following fluid administration denotes the development of iatrogenic hemodilution during spine surgery. SpHb and PVI traces obtained from the ROOT monitor, Masimo Inc., Irvine, CA, USA. Panel **a** reproduced with permission from Hospital Healthcare Europe (http://www.hospitalhealthcare.com/theatres/haemodilution-and-avoidable-blood-transfusions)

In one of the largest randomized controlled trials (RCTs) on GDT, the incidence of blood transfusions was double (22 vs 11%) in the GDT group patients, who received nearly twice the amount of colloids compared to the control group, even though the same transfusion threshold (Hb > 8 g/dl) was used for both groups [11]. The most feasible explanation for this clinically relevant and statistically significant difference (*p* = 0.04 based on a chi square test), which was not calculated nor discussed in the article [11], is that more patients in the GDT group reached Hb levels below the transfusion threshold due to hemodilution, prompting physicians to order blood transfusions. Other RCTs have also reported that patients in the GDT group, who received significantly more colloid boluses, received significantly more blood transfusions [12, 13] and had significantly higher blood loss [13] compared to the standard therapy group. In another study, the administration of more colloids led to lower Hb and DO2 values at the end of surgery (hemodilution being the responsible mechanism according to the authors themselves), and an associated trend of increased intraoperative blood loss [6]. In another prospective study comparing patients before and after the adoption of a GDT protocol, a pulse pressure variation-guided protocol was associated with less fluid administration, significantly higher Hb values after surgery, less blood transfusions, and decreased morbidity [14]. It seems, therefore, that the administration of greater amounts of fluids within a GDT protocol is frequently associated with more blood transfusions. It should be noted, however, that when fluid administration restores a depleted blood volume due to previous hemorrhage, the fall in Hb concentration may in fact reflect true (and not dilutional) anemia (Fig. 1a).

## Continuous non-invasive monitoring of hemoglobin (“SpHb”) may detect the development of iatrogenic hemodilution

Although questions have been raised regarding the absolute accuracy of continuous non-invasive measurement of Hb concentration (SpHb) [15], this parameter may be a useful trend monitor in the management of severe perioperative bleeding [2]. By offering real-time visibility of changes in Hb levels, SpHb monitoring may also detect real-time development of iatrogenic hemodilution in non-bleeding patients. Figure 1a and b depict trends from hepatic and spinal surgery, respectively, both known for significant changes in blood volume and frequent need for blood transfusions. In both cases, a significant decrease in SpHb to about 7.5 g/dL (dashed lines in Fig. 1a, b) is observed following the administration of large amounts of fluids. Simultaneously, there is a gradual and significant decrease in the Plethysmographic Variability Index (PVI), a dynamic parameter of fluid responsiveness, which is due to the increase in the effective blood volume. In some cases, the observed acute decrease in SpHb, in the absence of active bleeding, may help the clinician to reconsider the decision to administer blood transfusion. In addition, a gradual decrease in the SpHb in the absence of active bleeding should also prompt clinicians to re-examine their fluid management strategy.

## Conclusions

The administration of large amounts of intravenous fluids may cause iatrogenic hemodilution and, at times, even a paradoxical decrease in DO2. The associated decrease in Hb values to below the acceptable transfusion threshold may lead to avoidable blood transfusions. Perioperative GDT protocols that lead to the intentional administration of more fluids seem to be associated with more blood transfusions. By being able to reflect the development of acute iatrogenic hemodilution in real time, continuous monitoring of SpHb may be potentially helpful in identifying fluid overload and in the decision to transfuse blood.

## Abbreviations

GDT: Goal-directed therapy
Hb: Hemoglobin
PVI: Plethysmographic Variability Index
RCT: Randomized controlled trial
SpHb: Continuous non-invasively measured hemoglobin
